# Luminescence properties of silicon-cellulose nanocomposite

**DOI:** 10.1186/1556-276X-7-426

**Published:** 2012-07-31

**Authors:** Vitaly Pikulev, Svetlana Loginova, Valery Gurtov

**Affiliations:** 1 Department of Solid State Physics, Petrozavodsk State University, Petrozavodsk, 185640, Russia

**Keywords:** Nanosilicon, Porous silicon, Nanocellulose, Photoluminescence, Electret

## Abstract

We have characterized the structure and luminescence properties for two-component material composed of nanocrystalline cellulose and nanocrystalline (less to 100 nm) silicon powder. An efficient and stable photoluminescence of nanocomposite, resistant to the influence of gas-phase oxidants, has been found. The obtained material has electret-like properties and demonstrates the possibility of multiple-recharging in an electric field near 5·10^3^ V/cm at temperatures ranging from −70°C to 100°C. The presence of the electric field, as well as ozone or low-temperature plasma treatment, does not change the luminescence spectrum due to quantum size properties of silicon nanoparticles. We believe that these particles may appear in two states: both embedded in a cellulose matrix and in the form of mechanical mixture.

## Background

Luminescence properties of porous silicon or nanosilicon powder are severely degraded by the influence of gas-core oxidants (like O_2_, O_3_, and NO_2_) via oxidation of silicon nanoparticles [1,2]. To stabilize the photoluminescence (PL) activity of silicon nanoparticles, they are to be placed in an optically transparent medium (such as silica and polymer films). In the current case, the problem was solved by the introduction of silicon nanoparticles into a matrix of cellulose macromolecules. Despite the fact that polymer macromolecules synthesis with metal nanoparticles is rather thoroughly investigated [3], the studies of physical properties of silicon nanoparticles in natural polymers are poorly represented in scientific literature. We were able to find one paper [4] revealing phenomenon which is close to the current topic. In the above-mentioned work, the luminescent properties of ethyl cellulose nanoparticles containing embedded semiconductor nanocrystals have been investigated. Since only the general model of the luminescence mechanism was suggested by the author, the issue of the excitation transfer between the semiconductor nanoparticles and fragments of organic molecules is still under discussion. On the contrary, we are focused on the PL of silicon nanocrystals [5] in the solid-state media, since there is no generally accepted hypothesis to explain the phosphorescence of pure cellulose-I (without lignins) under ultraviolet (UV) excitation [6].

On the other hand, our choice of the matrix for the silicon nanoparticles injections is caused by the fact that cellulose is the most widespread and naturally renewable polymer. The development of environmentally friendly ‘paper electronics’ may bring the cost reduction in comparison with functional analogs of silicon electronics devices in the future. There is a reference for two papers: one of them describes the development of organic light-emitting diode based on luminescent cellulose [7] and the other presents the first ‘paper transistor’ on a carbon nanotube [8]. Currently, the technology of improvement of the physical properties of tissue (fire-resistant, in the first place) with silica nanoparticles is carried out into the industrial level of production [9]. We obtained the nanocomposite material which can be used currently as a hidden marker for identification of papers, showing stable luminescence properties under UV irradiation.

## Methods

There were three stages in the process of nanocomposite preparation. The method of preparation of nanocrystalline cellulose (NCC), which is based on a modification of chemically pure microcrystalline cellulose (MCC), pre-milled in an agate mortar in aqueous solution of 38% hydrochloric and 98% sulfuric acids (1:3:6 distilled water resp.) with periodic ultrasonic dispersion of mixture during several hours was used in the first stage of the experiment. The acidity of the solution was increased up to pH 3 by repeated dilution with distilled water. A similar method proposed in [10] allowed the authors to obtain particles of crystalline cellulose with diameter in the range from 20 to 100 nm (the results of transmission electron microscopy). In the current case, the value of the average size of a cellulose particle in suspension made up more than 60 nm, even after the separation of the heavy fraction of the suspension. This result can be explained by fast coagulation of the cellulose particles in the solutions. For further studies, we took samples from the upper (NCC-1) and lower (NCC-2) parts of the suspension which were subjected to partial sedimentation.

The second stage of the process consists of the preparation of nanocrystalline silicon. The source of silicon nanocrystals was porous silicon. For the preparation of porous Si, we used a standard electrochemical etching of p-Si wafer (100), 1 Ω·cm in 40% HF and isopropanol (1:1) solution by constant current density 50 mA/cm^2^ by visible light illumination. The suspension of particles of nanocrystalline silicon was formed in isopropanol solution by mechanical destruction of porous silicon sample placed in the solution. The silicon particles in the solution were subjected to subsequent ultrasonic dispersion of the colloidal solution for 30 min; as a result, the sizes of silicon particles in solution got distributed in the range from a few to 100 nm. In addition, a nonluminous Si powder, prepared by plasmachemical decomposition of monosilane in Ar plasma in an inductive discharge, was used.

The last stage consisted of mixing nanocellulose and Si nanoparticles in water-alcohol or pure alcohol solution by ultrasonic dispersion, evaporation, and molding by 0.26 MPa pressure. As a result a 0.2-mm thickness beige-colored tablet of nanocomposite with stable PL activity was obtained.

The X-ray investigations (X-ray diffraction, XRD) were performed on DRON-6.0 diffractometer (Bourevestnik, Inc., St. Petersburg, Russia) using CuKα-radiation. The degree of crystallinity of the original and the synthesized samples was carried out by X-ray data [11]. To obtain the infrared absorption spectra, the FT-801 Fourier transform infrared spectroscopy (FTIR) spectrometer (Simex, Inc., Novosibirsk, Russia) with nitrogen-cooled mercury cadmium telluride detector has been used. For the PL excitation we applied the 15 mW He-Cd laser.

## Results and discussion

**Figure 1:**
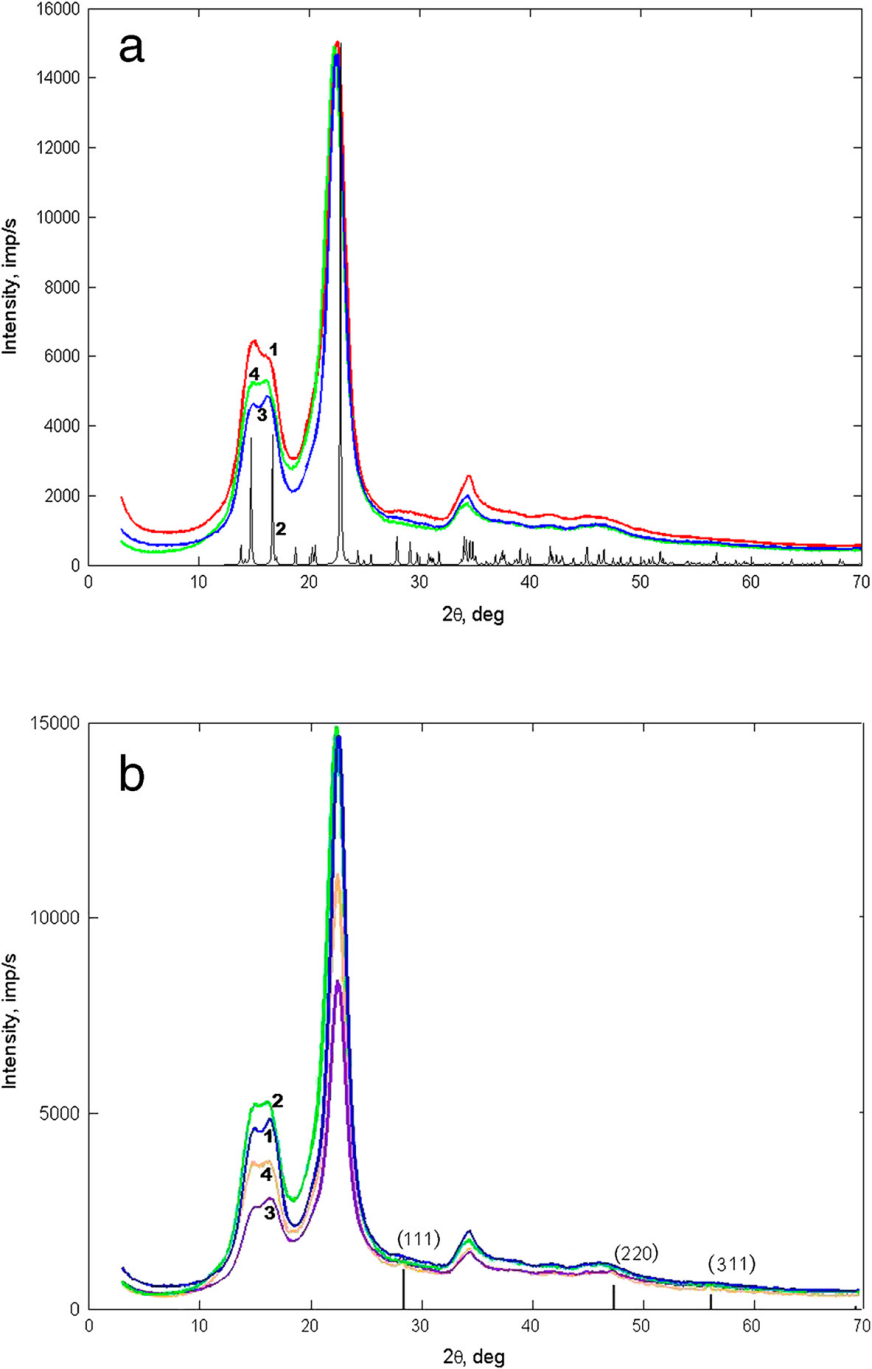
**XRD spectrum.** (**a**) MCC (1) compared with cellulose Iβ parallel up (2), NCC-1 (3) and NCC-2 (4). (**b**) Nanocomposite samples based on NCC-1 (3) and NCC-2 (4) compared with NCC-1 (1) and NCC-2 (2) and the data of Si (JCPDS 5–565).

**Figure 2:**
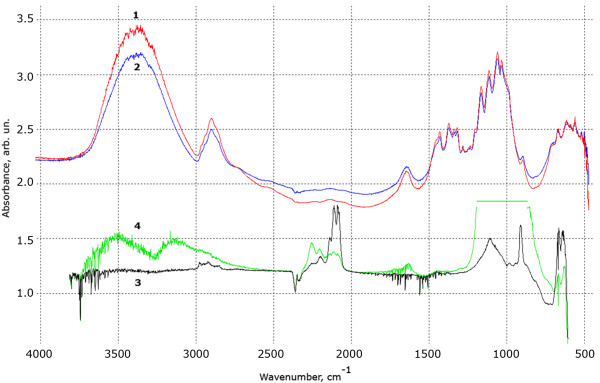
**FTIR spectra of nanocomposite.** (1) after 15 min of ozone treatment in comparison with pure MCC (2); as-prepared porous silicon and (3) porous silicon sample after similar ozone treatment (4).

X-ray diffraction patterns were obtained from MCC, nanocrystalline cellulose of two series (NCC-1 and NCC-2), and nanocomposites based on each series (Figure 1a,b). The figure shows that structure of MCC and NCC are similar to cellulose-Iβ ‘parallel up’, in space group Р2_1_[12]. The values of degree of crystallinity are MCC, 70% ± 3%; NCC-1, 78%; and NCC-2, 60%. The calculations performed on FTIR-spectra confirmed these values of degree of crystallinity. A typical FTIR-spectrum of our nanocomposites (Figure 2, curve 1) shows the positions of the absorption peaks, corresponding to the chemically pure cellulose-Iβ. The XRD analysis also showed a mean dimension of elemental fibril approximately 5 nm in each sample. 

There are weak peaks from the crystalline phase of silicon Si (Joint Committee on Powder Diffraction Standards - International Centre for Diffraction Data, JCPDS card 5–565) on X-ray diffraction pattern (Figure 1b). Since the amount of the amorphous phase in the first series of nanocomposite increases by 20% compared with the original NCC-1, we assume that during the synthesis of new material, a certain amount of silicon nanoparticles embedded in the cellulose matrix, and the rest formed mechanical mixture with it. In the second series of the composite, no increase on the amount of amorphous phase was observed. Thus, we conclude that the use of NCC with a lower degree of crystallinity makes possible a penetration the silicon nanoparticles in a polymer matrix without changing its structure.

**Figure 3:**
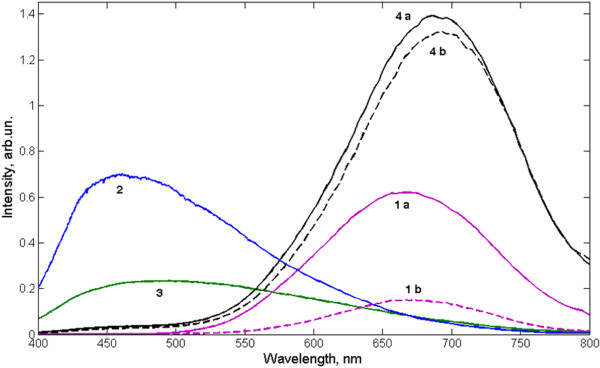
**Photoluminescence spectra**: 1, porous silicon (a - as-prepared, b - after 75 min of ozone treatment); 2, pure microcrystalline cellulose; 3, NCC; 4, and nanocomposite (a - as-prepared, b - after 75 min of ozone treatment).

All obtained nanocomposites, in contrast to a simple mechanical mixture of cellulose powder and porous silicon, exhibit bright PL in the energy band near 1.85 eV. To estimate the rate of degradation of the luminescent signals, ozone influence, ultraviolet radiation, low-temperature plasma torch, and heating the sample in atmosphere were used. In all cases the degree of exposure was chosen so that a property of cellulose had not been changed, while the degradation of PL of the freshly prepared porous silicon would have been obvious. Figure 2 shows that the short-time influence of ozone with concentrations above the maximum permissible concentration does not modify the chemical structure of the nanocomposite (curves 1 and 2), but significantly changes the infrared (IR) spectrum of porous silicon (curves 3 and 4). An increase in the intensity of the broad peak in the region 1,000 to 1,200 cm^−1^ indicates the growth of the silicon oxide phase. Reducing of the absorption peaks in 2,087, 2,116 and 2,140 cm^−1^ indicates a changing of hydrogen passivation on the surface of silicon nanoparticles [2]. Since cellulose molecules have a strong IR-absorption, the above-mentioned absorption lines of nano-Si in the composite do not appear on this background (curve 1 and 2). As a result of oxidation of silicon nanoparticles, luminescence properties of porous silicon is significantly reduced (see also Figure 3, curves 1 a and 1 b), that is not observed for our nanocomposite (Figure 3, curves 4 a and 4 b). 

The PL spectrum of nanocomposite (Figure 3, curve 4 a) shows more effective luminescence than as-prepared porous Si (curve 1 a) with an equal amount of nanomaterial. Curve 2 refers to PL of MCC sample. The shape of this spectrum in some cases split into several peaks, indicating the presence of few types of organic fluorophores in a matrix of cellulose. A possible reason for such luminescence might be the presence of carbonyl groups and different kinds of low-molecular products of cellulose destruction in this material [6]. After the formation of NCC, a fluorescent activity in short-wavelength region is significantly reduced (curve 3), and in the case of nanocomposite practically disappears (curve 4). In this case, the presence of Si nanoparticles leads to the suppression of short-wave emission, but it is premature to assume the existence of an energy transfer channel from cellulose matrix to semiconductor nanoparticles.

**Figure 4:**
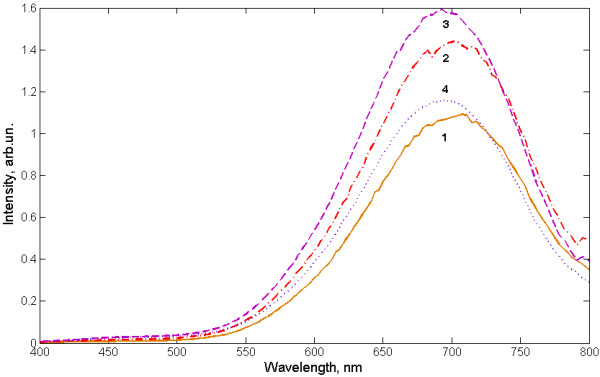
**Effect of low-temperature plasma treatment on the PL properties of the nanocomposite.** Curve 1, initial state of sample; 2, after 3 min; 3, after 6 min; and 4, after 12 min of plasma treatment.

The PL spectra of nanocomposite exposed in low-temperature plasma are shown in Figure 4. During the plasma exposure, the effect of enhancing the emission intensity for a main luminescence peak, followed by a minor degradation into initial value (the curves from 1 to 4) was observed. With a similar exposure for porous silicon, a short-term increasing of intensity, followed by irreversible quenching was detected. These results can be explained by the influence of the plasma torch, which burns in the atmosphere, on the stability of surface passivation of silicon nanoclusters in the cellulose matrix. The low-temperature plasma, in addition to ultraviolet irradiation, is a source of ozone, singlet oxygen, oxygen ions, nitrogen oxides, water, and OH-groups. In other words, the low-temperature plasma acts as a generator of different ion-exchange reactions on the surface of material. Since the oxidation of silicon nanocrystals is a common cause of degradation of the luminescence, we can assume primordial hydrogen passivation of Si nanoparticles in the composite. It also follows from the existence of a large number of hydrogen bonds between the cellulose macromolecules and crystallization water associated with cellulose nanoparticles (see Figure 2, 3,200 to 3,600 cm^−1^ water peak). Thus, the effect of low-temperature plasma for this material can be presented as a way to generate additional quantities of protons and hydroxyl ions, which improves the hydrogen passivation of silicon nanoparticles.

When a nanocomposite was heated in air, its PL properties were practically unchanged up to 100°C. Above this temperature the carbonization of the NCC begins, and the PL intensity greatly decreases. This fact is also consistent with the destruction of hydrogen passivation on the surface of nanocrystals due to breakdown of the cellulose molecules. Also note that dissolving of tablets in water and re-pressing remains the luminescent activity on the same level.

**Figure 5:**
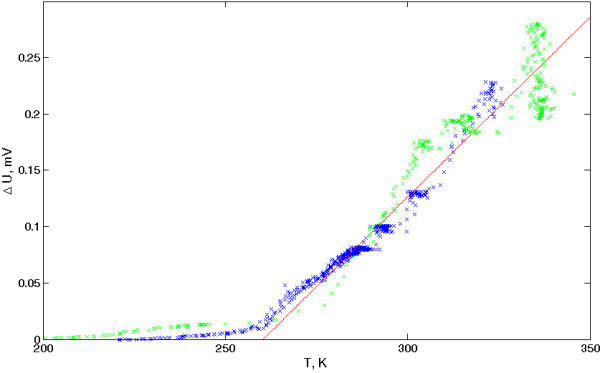
**Temperature dependence of the voltage between indium contacts to the sample during multiple cycles of ‘charging-freezing-heating’.** Points - experimental data, red line - empirical approximation.

Our silicon-cellulose nanocomposites exhibit electret-like behavior appearance of the residual voltage on the indium plates of the sample after charging during 10 min by constant voltage. After application of 200 V direct current (DC) (which corresponds to the electric field approximately 5·10^3^ V/cm), the initial residual voltage is approximately 1.2 V. The charge is partially restored after a short circuit and fully restored after recharging, with repeating polarity of the voltage source. Figure 5 shows the temperature dependence of the residual voltage for the sample, pre-charged during 10 min at 64 V DC. Several cycles of ‘charging-freezing-heating’ were performed. Since these effects were not detected close to liquid nitrogen temperature, it is possible to explain the results as a transfer of mobile ions in the cellulose matrix. Assuming a large number of protons and OH^–^-groups, the appearance of an ionic conductivity in this material is to be expected. It is also possible that the Si nanoparticles act as centers of charge localization. However, no influence of charge accumulation to luminescent properties of the nanocomposite was observed.

## Conclusions

Our presented results demonstrate a preparation method and specific PL and electrical properties of new ‘silicon-cellulose’ nanocomposite. In this case high retention of PL properties under external influences is determined for the ranges of chemical stability of cellulose molecules. The effects of temperature, gas-phase oxidation, and low-temperature plasma treatment show high stability of the PL properties of the composite. We have discussed a stabilization mechanism of the luminescent properties of nanocomposite, as well as the possibility of energy and charge transfer between the nanoparticles of silicon and cellulose.

## Abbreviations

DC,Direct current; FTIR,Fourier transform infrared spectroscopy; MCC,Microcrystalline cellulose; NCC,Nanocrystalline cellulose; PL,Photoluminescence; UV,Ultraviolet; XRD,X-ray diffraction.

## Competing interest

The authors declare that they have no competing interests.

## Authors’ contributions

VP prepared the samples, developed the experimental setup and provided PL and electrical measurements. SL prepared the samples and provided X-ray measurement. VG developed the concept of research and provides supervising of project. All authors participated in the interpretation of results. All authors read and approved the final manuscript.
